# Effectiveness of interventions to reduce indoor air pollution and/or improve health in homes using solid fuel in lower and middle income countries: protocol for a systematic review

**DOI:** 10.1186/s13643-015-0012-8

**Published:** 2015-03-04

**Authors:** Reginald Quansah, Caroline A Ochieng, Sean Semple, Sanjar Juvekar, Jacques Emina, Frederick Ato Armah, Isaac Luginaah

**Affiliations:** Biological, Environmental & Occupational Health Sciences, School of Public Health, College of Health Sciences, University of Ghana, Legon, Accra, Ghana; Department of Immunology, Noguchi Memorial Institute for Medical Research, College of Health Sciences, University of Ghana, Legon, Accra, Ghana; Stockholm Environment Institute, Stockholm, Sweden; Scottish Centre for Indoor Air Division of Applied Health Science, University of Aberdeen, Aberdeen, Scotland UK; KEM Hospital Research Centre, Pune, India; INDEPTH, Accra, Ghana; Department of Geography, Western University Canada, London, Ontario Canada

**Keywords:** Biomass, Indoor air pollution, Intervention, Protocol, Systematic review

## Abstract

**Background:**

Indoor air pollution (IAP) interventions are widely promoted as a means of reducing indoor air pollution/health from solid fuel use; and research addressing impact of these interventions has increased substantially in the past two decades. It is timely and important to understand more about effectiveness of these interventions. We describe the protocol of a systematic review to (i) evaluate effectiveness of IAP interventions to improve indoor air quality and/or health in homes using solid fuel for cooking and/or heating in lower- and middle-income countries, (ii) identify the most effective intervention to improve indoor air quality and/or health, and (iii) identify future research needs.

**Methods:**

This review will be conducted according to the National Institute for Health and Care Excellence (NICE) guidelines and will be reported following the PRISMA statement. Ovid MEDLINE, Ovid Embase, SCOPUS, and PubMed searches were conducted in September 2013 and updated in November 2014 (and include any further search updates in February 2015). Additional references will be located through searching the references cited by identified studies and through the World Health Organization Global database of household air pollution measurements. We will also search our own archives. Data extraction and risk of bias assessment of all included papers will be conducted independently by five reviewers.

**Discussion:**

The study will provide insights into what interventions are most effective in reducing indoor air pollution and/or adverse health outcomes in homes using solid fuel for cooking or heating in lower- or middle-income countries. The findings from this review will be used to inform future IAP interventions and policy on poverty reduction and health improvement in poor communities who rely on biomass and solid fuels for cooking and heating.

**Systematic review registration:**

The review has been registered with PROSPERO (registration number CRD42014009768).

**Electronic supplementary material:**

The online version of this article (doi:10.1186/s13643-015-0012-8) contains supplementary material, which is available to authorized users.

## Background

Over half of the world’s population is exposed to high concentrations of indoor air pollution (IAP) from the use of solid fuels for cooking and heating [1,2]. The resulting smoke from these fuels contains high concentrations of particulate matter (PM), carbon monoxide (CO), sulfur dioxides, nitrogen oxides, and other compounds known to be hazardous to human health [3-5]. Current epidemiological evidence suggests that indoor air pollution (IAP) from the use of solid fuel contributes importantly to the global burden of mortality and morbidity (for example, chronic obstructive pulmonary disease), accounting for about 1.6 million of the 59 million deaths annually [6-8]. In low-income countries, IAP from the use of solid fuel is now considered the top most risk factor for ill health: number one in South Asia and number two in sub-Saharan Africa [8]. In addition to health-damaging effects, solid fuel use has been identified as an important contributor to greenhouse gases (GHGs), ambient air pollution, deforestation, and environmental degradation [9].

Increasing number of interventions, including provision of clean cookstoves, installation of chimneys and hoods, and behavioral measures such as health education have been carried out in the last two decades to reduce IAP from solid fuel use [10-13]. However, there has been no attempt to systematically synthesize the evidence that IAP interventions in homes using solid fuel improve indoor air quality/health. It is therefore timely and important to understand more about the effectiveness of these interventions. In this paper, we describe the protocol of a systematic review to (i) evaluate the effectiveness of IAP interventions to improve indoor air quality and/or health in homes using solid fuel for cooking and heating in lower- and middle-income countries, (ii) identify the most effective intervention to improve indoor air quality and/or health, and (iii) identify future research needs.

## Methods

We will develop this systematic review according to established methods, based on those used by the National Institute for Health and Care Excellence (NICE) [14] and the National Academy of Science review of the EPA Integrated Risk Information System (IRIS) process [15]. We will report the findings according to recommendations from the Preferred Reporting Items for Systematic Reviews and Meta-Analyses statement (PRISMA) [16]. We will develop a comprehensive database containing all published studies on IAP interventions that report on indoor air quality and/or health outcome. The purpose of this comprehensive database is to build and critically analyze global evidence on this subject as part of a proposed collaborative project among the School of Public Health, University of Ghana (represented by RQ), Stockholm Environment Institute, Sweden (represented by CO), INDEPTH, Ghana (represented by JE and SJ), Scottish Centre for Indoor Air Division of Applied Health Science, UK (represented by SS), and the University of Ontario (represented by IL and FAA). This review is registered with the International Prospective Register of Systematic Reviews (PROSPERO) (http://www.crd.york.ac.uk/PROSPERO/display_record.asp?ID=CRD42014009768#.VO7yzi4YFTs) and has the registration number CRD42014009768 allocated to it.

### Search strategy

We will identify search terms by extracting key terms from reviews and selected relevant papers and review Medical Subject Headings for relevant and appropriate terms. In order to ensure adequate sensitivity of the search strategy, RQ piloted the search in PubMed with the search terms and keywords (searched 10 October 2013). This resulted in 28,437 hits, of which all ten relevant papers [10,17-25] that we had identified prior to running the search, were included. RQ will search the following databases: Ovid MEDLINE, Ovid Embase, SCOPUS, and PubMed. The searches were conducted in September 2013 and updated in November 2014. We will also update the search in February 2015. The rest of the research team will search through WHO Global database of household air pollution measurements [26] and members’ archives. All references will be imported into Reference Manager. Search terms/keywords that will be used to identify relevant studies is attached as Additional file 1.

### Inclusion and exclusion criteria

#### Types of study

All randomized controlled trials (RCTs and quasi-RCTs) and all non-randomized control trials (that is, cohort, case-controlled, and cross-sectional studies) and controlled before-and-after studies conducted in low- or middle-income countries will be included. Definition of low or middle income is based on the United Nations Human Development Report released in March 2013 [27]. We are aware that the position of countries in such indices changes over time, and we will update our definition accordingly. We will exclude all studies not conducted in homes and studies conducted in developed countries.

#### Type of intervention

We will be interested in any type of household intervention that is explicitly aimed at improving indoor air quality and/or health from solid fuel use. Such interventions will include, for example, changes in stove or heating apparatus, changes in ventilation arrangements, and changes in behavior geared towards reducing emission and exposure to cooking smoke. Interventions targeting, for example, deforestation, fire wood use, particle size distribution, and cooking time will be excluded. Intervention as used in this protocol is defined as a deliberate measure employed at household or community level with a goal of long-/short-term reduction in exposure or health effects associated with IAP exposure from solid fuel use.

#### Population of interest

The review will include all interventions on women and children specifically, as well as the more general population.

#### Type of comparisons

There will be no restrictions on the type of comparator used in the intervention study (for example, convenience comparison group, randomized control group, no intervention control, and usual practice control). That is, studies with and without comparators will be included in the review.

#### Types of publications

We will consider both articles in peer-reviewed journals and student theses. There will be no language restrictions, and, where possible, literature in languages other than English will be translated. We will report any literature which we are unable to translate.

#### Types of outcome

All measured outcomes indicative of indoor air quality and health outcomes will be included. Measures of air quality may, for example, include (but not be limited to) airborne concentrations of carbon monoxide or fine particulate matter. Common health indicators reported may include (but again not be limited to) acute lower respiratory infection, sensory irritation (for example, itchy/watery/sore eyes), cough, high blood pressure, and so on (see Additional file 2). Studies that only report on fuel use, cooking time, climate, and non-indoor air quality/health related outcomes will be excluded.

#### Data extraction and risk of bias assessment

Initial screening of titles and abstracts retrieved from the searches will be conducted by three reviewers (RQ, SS, and CO) to ensure consistency. Full papers of potentially relevant publications will be located and independently appraised by five reviewers (RQ, CO, SS, FA, and IL) to select those satisfying the inclusion criteria. Data extraction of all included papers will be conducted independently by the above authors (that is, RQ, CO, SS, FA, and IL). Throughout, any discrepancies will be resolved through discussion. When the reported data are insufficient, RQ will contact the corresponding authors by e-mail to request additional information.

An electronic data extraction form applied in a previous study [28] will be modified and used (Additional file 3). Data to be extracted will include general study information (author name, title, source, country of origin, reviewer identification, study identification, and funding source), study characteristics (study design, population targeted, demographics, recruitment and follow-up rates, and so on), health outcome(s), exposures of interest, method of analysis (*a priori* power calculation, statistical analysis, confounding factors, and subgroup analysis), and results. Risk of bias will be assessed with Effective Public Health Practice Project Quality Assessment Tool for Quantitative Studies (EPHPP) [29] (see Additional file 4A, B) by RQ, CO, SS, FA, and IL. EPHPP focuses on six domains: selection bias, study design, confounders, blinding, data collection, and withdrawals and dropouts. We will grade each study as strong risk of bias, moderate risk of bias, and weak/low risk of bias.

#### Analysis and synthesis

We will analyze our findings following the Economic and Social Research Council (ESRC) Narrative Synthesis Guidance [30]. Interventions will be grouped according to population type (children, women, and the general population) and outcomes. Our qualitative data will be synthesized thematically. For few studies reporting on the same outcome, we will compute the overall summary effect estimates and no further analysis will be carried out. We plan also to match qualitative findings with quantitative findings using a method proposed by Evidence for Policy and Practice Information and Co-ordinating Centre [31].

### Presenting and reporting of results

We plan to use flow diagrams to summarize our study selection process and reasons for exclusion. This will follow PRISMA guidelines for reporting systematic reviews [32]. We will also use graphical tables to summarize findings from individual studies [33]. We will append our search strategy and individual study risk of bias assessment as supplemental materials.

## Discussion

This systematic review is aimed at synthesizing evidence on the effectiveness of household interventions to improve indoor air quality and/or health in low- to middle-income countries. Our pilot search identified all ten studies we identify prior to running our search. The strength of this review is that we will be able to examine different types of IAP interventions and provide insights into what interventions are most effective in reducing indoor air pollution and adverse health outcomes. The findings from this review will be used to inform future policy and programs aimed at reducing the high health burden of IAP exposure from solid fuel use.

## Additional files

Additional file 1:
**Search terms/keywords that will be used to identify studies for the review.**
Additional file 2:
**Selected outcomes considered in the protocol.**
Additional file 3:
**Data extraction form - cohort study.**
Additional file 4:
**A. Design-specific criteria to assess for risk of bias with the Effective Public Health Practice Project Quality Assessment Tool for Quantitative Studies (EPHPP).** **B**. Global Rating of the Effective Public Health Practice Project Quality Assessment Tool for Quantitative Studies (EPHPP) score.

## Abbreviations

CO: carbon monoxide
EPHPP: Effective Public Health Practice Project Quality Assessment Tool for Quantitative Studies
ESRC: Economic and Social Research Council
GHGs: contributor to greenhouse gases
IAP: indoor air pollution
IPCC: Intergovernmental Panel on Climate Change
IRIS: Integrated Risk Information System
NICE: National Institute for Health and Care Excellence
PM: particulate matter
PRISMA: Preferred Reporting Items for Systematic Reviews and Meta-Analyses statement
WHO: World Health Organization
