# Supplementary material for: Effectiveness of interventions to reduce indoor air pollution and/or improve health in homes using solid fuel in lower and middle income countries: protocol for a systematic review
Source: Syst Rev. 2015 Mar 4;4:22. doi: 10.1186/s13643-015-0012-8 (PMC4378274; doi:10.1186/s13643-015-0012-8)
Supplement: Additional file 1: — Search terms/keywords that will be used to identify studies for the review. [file 13643_2015_12_MOESM1_ESM.doc]

**Additional file 1**. Search terms/keywords that will be used to identify studies for the review

| **Keywords/search terms** | |
| --- | --- |
| 1. Stove* 2. Biomass fuel 3. IAP 4. Indoor pollu* 5. Carbon mono-oxide 6. Solid fuel 7. Crop residue 8. Dung 9. Improve fuel 10. Biomass stove 11. Charcoal 12. Particulate matter 13. Household air pollu* 14. improved ventilation 15. Biofuel 16. Inhalation Exposure 17. Heating 18. Coal 19. Heating fuel 20. Cooking fuel 21. Wood smoke 22. Improved stoves 23. Cleaner stove 24. Cook* smoke 25. Pollutant 26. Air quality 27. 1 OR 2 OR 3 OR 4……0R 26 | 1. Intervention 2. Program evaluation 3. Behavior change 4. Behaviour change 5. Environmental monitoring 6. Experimental group 7. Randomized 8. Controlled trial 9. Controlled study 10. Randomized control 11. Health education 12. Health behaviour 13. Health behaviour 14. Comparison group 15. Control group 16. Hood 17. 28 OR 29 OR 30 OR 31……..OR 43 |
| Combined   1. 27 AND 44 | |
