# Supplementary material for: Effectiveness of interventions to reduce indoor air pollution and/or improve health in homes using solid fuel in lower and middle income countries: protocol for a systematic review
Source: Syst Rev. 2015 Mar 4;4:22. doi: 10.1186/s13643-015-0012-8 (PMC4378274; doi:10.1186/s13643-015-0012-8)
Supplement: Additional file 2: — Selected outcomes considered in the protocol. [file 13643_2015_12_MOESM2_ESM.doc]

**Additional file 2.** Selected Outcomes considered in the protocol

| **Outcome** | |
| --- | --- |
| **Exposure** | **Health** |
| Carbon monoxide | Pneumonia |
| Particulate matter (PM2.5) | Asthma |
| Polycyclic aromatic hydrocarbon | Eye disease |
| Air quality | Cataract |
|  | COPD |
|  | Cardiovascular diseases |
|  | Tuberculosis |
|  | Pulmonary disease |
|  | Lung cancer |
|  | Cancer |
|  | Hypertension |
|  | Infant mortality |
|  | High blood pressure |
