# Supplementary material for: Effectiveness of interventions to reduce indoor air pollution and/or improve health in homes using solid fuel in lower and middle income countries: protocol for a systematic review
Source: Syst Rev. 2015 Mar 4;4:22. doi: 10.1186/s13643-015-0012-8 (PMC4378274; doi:10.1186/s13643-015-0012-8)
Supplement: Additional file 3: — Data extraction form - cohort study. [file 13643_2015_12_MOESM3_ESM.doc]

**Additional file 3. Data extraction form- Cohort study**

| *Full paper eligibility for review* |  |
| --- | --- |
| **General information** |  |
| Date of data extraction |  |
| Author |  |
| Article title |  |
| Source (Year /Journal / Volume/ Pages) |  |
| Country of origin |  |
| Type of publication |  |
| Identification of reviewer |  |
| Notes |  |
| Identification number |  |
| **Specific information** |  |
| *Methodological quality of study* |  |
| Study design |  |
|  |  |
| ***Population characteristics*** |  |
| ***Cohort*** |  |
| Source of population |  |
| Inclusion criteria |  |
| Exclusion criteria |  |
| Recruitment procedures used |  |
| Total number of participants eligible/selected/contacted for study data collection and randomisation |  |
| Total number of participants recruited in the intervention group |  |
| Total number of participants recruited in the Control group |  |
| Total number of participants responded/agreed to take part in the intervention group |  |
| Total number of participants responded/agreed to take part in the control group |  |
| Number at follow up/Loss to follow up/Drop out rate in the intervention group |  |
| Number at follow up/Loss to follow up/Drop out rate in the control group |  |
| State characteristics of intervention (I) and Control (C) groups   - Age - Sex - Geographical region - Socio-economic status - Ethnicity - High risk (family history)   Others |  |
| - Ethnicity - High risk (family history) - Others |  |
| Exposure cohort |  |
| Methods of follow up |  |
| Follow up time cases/follow up duration |  |
| ***Assessments*** |  |
| **Health** |  |
| **Exposures of interest** |  |
| **List exposure(s) of interest (intervention)** |  |
| objective assessed in the study |  |
| Repeated personal exposure sampling with each sample >24 hours. |  |
| Repeated personal exposure sampling with each sample <24 hours. |  |
| Single personal exposure sampling > 24 hours. |  |
| Single personal exposure sampling < 24 hours. |  |
| Repeated biological monitoring data for IAP metabolite |  |
| Single biological monitoring data for IAP or IAP metabolite |  |
| Repeated area/static exposure sampling from several household locations |  |
| Single area/static exposure sampling from several household locations |  |
| Repeated area /static exposure sampling from one household location |  |
| Single area /static exposure sampling from one household location |  |
| Self-report of IAP producing activity with data on exposure modifiers and some method of quantification or semi-quantification |  |
| Self-report of presence/absence of IAP producing activity |  |
| Proxy-respondent report of either of the above two methods. |  |
|  |  |
| ***Analysis*** |  |
| A priori power calculation |  |
| Method of analysis: statistical analysis used |  |
| Potential confounding factors |  |
| Subgroup analysis |  |
| Missing data + how addressed |  |
|  |  |
| ***Results*** |  |
| General |  |
| Primary outcome |  |
| Other outcomes |  |
| **Extra useful information** |  |
| Limitations |  |
| Interpretation |  |
| Generalisibility |  |
| Is the methodology appropriate to provide valid and reliable answers to the study question/s? |  |
| Funding |  |
| Other |  |
