# Supplementary material for: Effectiveness of interventions to reduce indoor air pollution and/or improve health in homes using solid fuel in lower and middle income countries: protocol for a systematic review
Source: Syst Rev. 2015 Mar 4;4:22. doi: 10.1186/s13643-015-0012-8 (PMC4378274; doi:10.1186/s13643-015-0012-8)
Supplement: Additional file 4: — A. Design-specific criteria to assess for risk of bias with the Effective Public Health Practice Project Quality Assessment Tool for Quantitative Studies (EPHPP). B. Global Rating of the Effective Public Health Practice Project Quality Assessment Tool for Quantitative Studies (EPHPP) score. [file 13643_2015_12_MOESM4_ESM.doc]

**Additional file 4A.** Design-specific criteria to assess for risk of bias with the Effective Public Health Practice Project Quality Assessment Tool for Quantitative Studies (EPHPP)

| **Risk of bias** | **Criterion** | **Rating** |
| --- | --- | --- |
| A. Selection bias | 1.Are the individuals selected to participate in the study likely to be representative of the target population? | 1. Very likely 2. Somewhat likely 3. Not likely 4. Can’t tell (no information on participation rate |
|  | 2. What percentage of selected individuals agreed to participate? | 1. 80 - 100% agreement 2. 60 – 79% agreement 3. less than 60% agreement 4. Not applicable   5.Can’t tell |
| B. Study design | 1.Indicate the study design | 1. Randomized controlled trial 2. Controlled clinical trial 3. Cohort analytic (two group pre + post) 4. Case-control 5. Cohort (one group pre + post (before and after)) 6. Interrupted time series 7. Cross-sectional 8. Other specify ____________________________ |
|  | 2. Was the study described as randomized? If NO, go to Component C. | 1.No  2.Yes |
|  | 3.If Yes, was the method of randomization described? (See dictionary) | 1.No  2.Yes |
|  | 4. If Yes, was the method appropriate? (See dictionary) | 1.No  2.Yes |
| C. Confounders | 1.Were there important differences between groups prior to the intervention? | 1. Yes 2. No 3. Can’t tell |
|  | 2. If yes, indicate the percentage of relevant confounders that were controlled (either in the design (e.g. stratification, matching) or analysis)? | 1. 80 – 100% (most) 2. 60 – 79% (some) 3. Less than 60% (few or none)   4. Can’t Tell |
| D. Blinding | 1.Was (were) the outcome assessor(s) aware of the intervention or exposure status of participants? | 1. Yes 2. No 3. Can’t tell |
|  | 2. Were the study participants aware of the research question? | 1. Yes 2. No 3. Can’t tell |
| E. Data collection methods | 1. Were data collection tools shown to be valid? | 1. Yes 2. No   3.Can’t tell |
|  | 2. Were data collection tools shown to be reliable? | 1. Yes 2. No   4. Can’t tell |
| F. Withdrawals and drop-outs | 1. Were withdrawals and drop-outs reported in terms of numbers and/or reasons per group? | 1. Yes 2. No 3. Can’t tell   4.Not Applicable |
|  | 2. Indicate the percentage of participants completing the study. (If the percentage differs by groups, record the lowest). | 1. 80 -100% 2. 60 - 79% 3. less than 60% 4. Can’t tell 5. Not applicable |

**Additional file 4B**. Global Rating of the Effective Public Health Practice Project Quality Assessment Tool for Quantitative Studies (EPHPP) score.

| **Risk of bias** | **Rating** | | | |
| --- | --- | --- | --- | --- |
|  | Strong | Moderate | Low | Not Applicable |
| A. Selection bias |  |  |  |  |
| B. Study design |  |  |  |  |
| C. Confounders |  |  |  |  |
| D. Blinding |  |  |  |  |
| E. Data collection methods |  |  |  |  |
| F. Withdrawals and drop-outs |  |  |  |  |
